# Improved survival outcomes and restoration of graft‐vs‐leukemia effect by deferasirox after allogeneic stem cell transplantation in acute myeloid leukemia

**DOI:** 10.1002/cam4.1928

**Published:** 2019-01-24

**Authors:** Byung‐Sik Cho, Young‐Woo Jeon, A‐Reum Hahn, Tai‐Hyang Lee, Sung‐Soo Park, Jae‐Ho Yoon, Sung‐Eun Lee, Ki‐Seong Eom, Yoo‐Jin Kim, Seok Lee, Chang‐Ki Min, Seok‐Goo Cho, Jong‐Wook Lee, Woo‐Sung Min, Hee‐Je Kim

**Affiliations:** ^1^ Division of acute leukemia, Catholic Hematology Hospital, Seoul St. Mary's Hospital The Catholic University of Korea Seoul Korea; ^2^ Leukemia Research Institute, College of Medicine The Catholic University of Korea Seoul Korea

**Keywords:** acute myeloid leukemia, allogeneic hematopoietic stem cell transplantation, deferasirox, graft‐vs‐leukemia effects, hyperferritinemia

## Abstract

Deferasirox is an oral iron‐chelating agent having possible antileukemia and immune modulatory effects. Few reports have evaluated deferasirox in the setting of allogeneic hematopoietic stem cell transplantation (allo‐HSCT). We investigated the impact of deferasirox after allo‐HSCT in acute myeloid leukemia (AML). Of 326 consecutive patients undergoing allo‐HSCT in remission, analysis of 198 patients not receiving deferasirox revealed the negative prognostic effect of hyperferritinemia (≥1000 ng/mL) before and after allo‐HSCT on survival mainly due to increase in relapse. Of 276 patients with hyperferritinemia at 1 month after allo‐HSCT, 128 patients (46%) received deferasirox. Deferasirox induced a faster decline in serum ferritin level with a manageable safety profile, which significantly reduced relapse rather than nonrelapse mortality, resulting in better survival compared to patients not receiving deferasirox. Of note, the deferasirox group had a significantly higher incidence of chronic graft‐vs‐host disease, indicating improved graft‐vs‐leukemia (GVL) effects evidenced by the presence of suppressed regulatory T cells and sustained higher proportion of NK cells in peripheral blood. This study firstly demonstrates the improved survival and restoration of GVL effects of patients with AML by deferasirox, which also clarifies the detrimental effect of hyperferritinemia through after allo‐HSCT.

## 1 INTRODUCTION

Elevated serum ferritin (SF) is commonly found after allogeneic hematopoietic stem celltransplantation (allo‐HSCT), which is the easiest and most useful methods of determining iron overload.^1^, ^2^, ^3^ However, SF is an imperfect surrogate measure of iron stores as inflammation and cancer could affect SF levels.^3^ Some studies recommended the liver iron content to define iron overload instead of SF.^4^, ^5^ Several lines of evidence have demonstrated that ferritin is a multifunctional protein, involved in cell proliferation, angiogenesis, and immunosuppression, as well as iron delivery,^6^ which turns SF into an attractive candidate of biomarker for patients with allo‐HSCT potentially reflecting the status of iron stores, inflammation, and tumor burden. Indeed, a meta‐analysis showed that liver iron contents itself failed to predict allo‐HSCT outcomes,^4^ while hyperferritinemia was a significant prognostic factor.^4^, ^5^

Several studies have analyzed the role of hyperferritinemia in affecting the outcomes of allo‐HSCT.^5^, ^7^, ^8^, ^9^, ^10^, ^11^, ^12^, ^13^, ^14^ Recent meta‐analyses reported that elevated SF level at pre‐transplantation is related to a low overall survival (OS) and high nonrelapse mortality (NRM)^15^, ^16^ and pre‐transplantation iron chelation may be associated with improved survival and reduced NRM.^17^ On the other hand, there exist only a few reports on the role of hyperferritinemia after allo‐HSCT suggesting possible significant association with decreased survival.^18^, ^19^ Based on the suggestive detrimental role of hyperferritinemia after allo‐HSCT, therapeutic strategies to reduce SF levels after allo‐HSCT seem to be rational. Deferasirox, an oral iron‐chelating agent having possible antileukemia^20^, ^21^, ^22^ and immune modulatory effects,^23^, ^24^, ^25^ is a best candidate. Recent small prospective studies demonstrated a manageable safety profile and efficacy of deferasirox, in the setting of allo‐HSCT.^26^, ^27^ However, the impact of deferasirox after allo‐HSCT on the survival of AML patients is not yet known. In this study, we evaluate the prognostic role of hyperferritinemia not only before and but also after allo‐HSCT in a homogeneous disease group of AML patients. In addition, the impact of deferasirox after allo‐HSCT on long‐term survival outcomes was determined by comparing the outcomes of patients not receiving deferasirox.

## 2 MATERIALS AND METHODS

### 2.1 Patients

**Figure 1:**
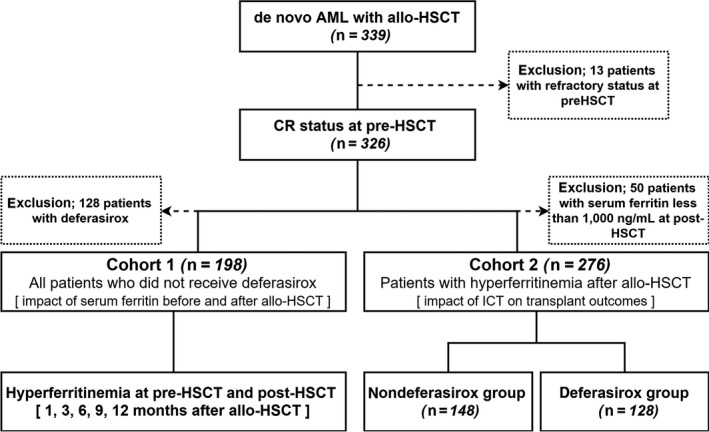
Study design. AML, acute myeloid leukemia; CR, complete remission; HSCT, hematopoietic stem cell transplantation

We retrospectively evaluated 339 consecutive patients diagnosed with de novo AML, who underwent unmanipulated allo‐HSCT at Catholic Blood and Marrow Transplantation Center between January 2007 and February 2012. Thirteen patients with refractory disease at allo‐HSCT were excluded in this study. Finally, 326 patients with complete remission (CR) at allo‐HSCT were analyzed (Figure 1). Table 1 lists the demographic information of all the enrolled patients. The median age of patients was 41 years (range, 18‐66 years), and 172 patients were male gender (52.8%). Patients transplanted from matched siblings (54.9%), unrelated (33.7%), and haploidentical‐related donors (11.3%) received myeloablative (65.3%) or reduced intensity conditioning (34.7%). Stem cell sources were bone marrow (48.2%) and peripheral blood (51.8%), and antithymocyte globulin (ATG; thymoglobulin; Genzyme, Cambridge, MA) was added in graft‐vs‐host disease (GVHD) prophylaxis for patients transplanted from unrelated and haploidentical donors.^28^ Treatment courses and transplantation procedure were performed as previously described.^29^ The Catholic Medical Center Institutional Review Board approved this single‐center study; all the analyses were performed following Institutional Review Board guidelines and the rules of the Declaration of Helsinki.

**Table 1:** Patients’ characteristics

Factors	All patients (n = 326)	Cohort 1				Cohort 2			
		Total (n = 198)	Low SF (n = 94)	High SF (n = 104)	*P*	Total (n = 276)	Non‐ICT (n = 148)	ICT (n = 128)	*P*
Age at diagnosis, median(range)	41 (18‐66)	41 (19‐66)	40 (19‐66)	41 (19‐64)	0.416	42 (18‐66)	40 (31‐50)	45 (36‐66)	**0.047**
Gender (%)									
Male	172 (52.8)	98 (49.5)	39 (41.5)	59 (56.7)	**0.046**	172 (52.8)	79 (53.4)	74 (57.8)	0.537
Female	154 (48.2)	100 (50.5)	55 (58.5)	45 (43.3)		154 (47.2)	69 (46.6)	54 (42.2)	
Risk by ELN (%)									
Good	39 (12.0)	24 (12.1)	14 (14.9)	10 (9.6)	0.072	39 (12.0)	16 (10.8)	15 (11.7)	0.089
Intermediate‐1	146 (44.8)	83 (41.9)	44 (46.8)	39 (37.5)		146 (44.8)	56 (37.8)	63 (49.2)	
Intermediate‐2	78 (23.9)	45 (22.7)	14 (14.9)	31 (29.8)		78 (23.9)	40 (27.0)	33 (25.8)	
Poor	63 (19.3)	46 (23.2)	22 (23.4)	24 (23.1)		63 (19.3)	36 (24.3)	17 (13.3)	
Serum ferritin at diagnosis, median (range)	1117 (10‐7455)	1075 (10‐7455)	832 (10‐7455)	1294 (175‐6880)	**0.005**	776 (395‐1535)	879 (474‐1289)	808 (395‐1535)	0.492
Serum ferritin at diagnosis,^a^ median (range)	859 (5‐3070)	809 (10‐3050)	566 (10‐2611)	1043 (176‐3050)	**<0.001**	919 (5‐3070)	906 (10‐3050)	931 (5‐3070)	0.786
Serum ferritin at pre‐HSCT, median (range)	1462 (132‐12 386)	1383 (132‐12 386)	584 (132‐989)	2148 (1000‐12 386)	—	1130 (646‐1780)	1210 (775‐1985)	1219 (819‐1851)	0.677
Transfusion, median (range)									
Packed RBC unit	17 (0‐56)	16 (0‐56)	13 (0‐32)	19 (0‐56)	**<0.001**	16 (10‐22)	16 (12‐22)	16 (11‐24)	0.519
Platelet (SDP) unit	22 (0‐103)	21 (0‐103)	17 (0‐60)	25 (0‐103)	**<0.001**	18 (11‐29)	19 (12‐30)	18 (11‐31)	0.710
Total unit	39 (0‐171)	39 (0‐171)	31 (0‐83)	45 (0‐171)	**<0.001**	34 (23‐50)	36 (24‐52)	36 (24‐56)	0.746
Stem cell source (%)									
Bone marrow	157 (48.2)	53 (56.4)	53 (56.4)	91 (87.5)	0.830	125 (45.3)	77 (52.0)	48 (37.5)	**0.022**
Peripheral blood	169 (51.8)	41 (43.6)	5 (5.3)	13 (12.5)		151 (54.7)	71 (48.0)	80 (62.5)	
Donor type (%)									
Sibling	179 (54.9)	116 (58.6)	56 (59.6)	60 (57.7)	0.839	147 (53.3)	84 (56.8)	63 (49.2)	**<0.001**
Unrelated	110 (33.7)	74 (37.4)	35 (37.2)	39 (37.5)		94 (34.1)	57 (38.5)	37 (28.9)	
Haploidentical	37 (11.3)	8 (4.0)	3 (3.2)	5 (4.8)		35 (12.7)	7 (4.7)	28 (21.9)	
Conditioning intensity (%)									
Myeloablative	213 (65.3)	143 (72.2)	70 (4.5)	73 (70.2)	0.609	176 (63.8)	106 (71.6)	70 (54.7)	**0.005**
Reduced intensity	113 (34.7)	55 (27.3)	24 (25.5)	31 (29.8)		100 (36.2)	42 (28.4)	58 (45.3)	
ATG usage (%)									
No	207 (63.5)	133 (67.2)	65 (69.1)	68 (65.4)	0.681	169 (61.2)	95 (64.2)	74 (57.8)	0.337
Yes	119 (36.5)	65 (32.8)	29 (30.9)	36 (34.6)		107 (38.8)	53 (35.8)	54 (42.2)	
GVHD prophylaxis (%)									
Cyclosporin with MTx.	179 (54.9)	115 (58.1)	55 (58.5)	60 (57.7)	0.980	147 (53.3)	83 (56.1)	64 (50.0)	**<0.001**
Tacrolimus with MTX	147 (45.1)	83 (41.9)	39 (41.5)	44 (42.3)		129 (46.7)	65 (43.9)	64 (50.0)	
Donor age, median (range)	35 (7‐67)	35 (7‐65)	35 (7‐60)	36 (8‐65)	0.427	36 (7‐67)	36 (7‐65)	36 (8‐67)	0.848
Donor gender (%)									
Male	194 (59.5)	121 (61.1)	58 (61.7)	63 (60.6)	0.987	161 (58.3)	88 (59.5)	73 (57.0)	0.775
Female	132 (40.5)	77 (38.9)	36 (38.3)	41 (39.4)		115 (41.7)	60 (40.5)	55 (43.0)	
ABO‐matched status (%)									
Match	179 (54.9)	110 (55.6)	51 (54.3)	59 (56.7)	0.836	154 (55.8)	85 (57.4)	69 (53.9)	0.641
Mismatch	147 (45.1)	88 (44.4)	43 (45.7)	45 (43.3)		122 (44.2)	63 (42.6)	59 (46.1)	
HLA‐matched status (%)									
Match	249 (76.4)	162 (81.8)	77 (81.9)	85 (81.7)	0.984	207 (75.0)	120 (81.1)	87 (68.0)	**0.018**
Mismatch	77 (23.6)	36 (18.2)	17 (18.1)	19 (18.3)		69 (25.0)	28 (18.9)	41 (32.0)	
CD34+ cell dose (×10^6^/kg), median (range)	4.7 (0.9‐16.9)	4.7 (0.9‐12.9)	4.7 (0.9‐14.1)	4.7 (1.1‐12.9)	0.887	4.7 (2.5‐16.2)	4.7 (2.5‐16.9)	4.6 (2.8‐16.2)	0.370

The bold values represent the statistical significance.

ATG, antithymocyte globulin; ELN, European Leukemia Net; GVHD, graft‐vs‐host disease; ICT, iron‐chelating therapy; MTx, methotrexate; SDP, single donor platelet; SF, serum ferritin.

a Patients who did not have concurrent infections and previous transfusions.

### 2.2 Iron‐chelating therapy (ICT) with deferasirox and study design

The SF levels were monitored from the time of initial diagnosis of AML to the several time points at the pre‐ and post‐transplantation period (1, 3, 6, 9, and 12 months after allo‐HSCT). C‐reactive protein levels were simultaneously measured along with SF, which was almost within the normal range with a slight increase at pre‐transplantation and 1 month after allo‐HSCT (Figure S1). Patients who developed hyperferritinemia (≥1000 ng/mL) at least a month after transplantation were recommended deferasirox administration (10‐20 mg/kg/d). If agreed without contraindications, such as elevated serum creatinine level >2 times the upper normal limit, low platelet counts <50 × 10^9^/L, known hypersensitivity to deferasirox, poor performance status, and refusal, deferasirox was started and continued until the ferritin level was below 500 ng/mL and unless there occurred any serious adverse events and/or relapse.

This study was designed to separately evaluate two cohorts (Figure 1). The evaluation of the first cohort including patients not receiving deferasirox (n = 198) was aimed to assess the impact of hyperferritinemia before and after allo‐HSCT on transplantation outcomes. The evaluation of second cohort consisting of patients who had hyperferritinemia at 1 month after allo‐HSCT (n = 276) was aimed to assess the safety and efficacy of deferasirox compared to the nondeferasirox group. In the second cohort, 128 patients (46%) received deferasirox, while 148 patients (54%) were not administered deferasirox due to patients’ disagreement (92%) or contraindications for deferasirox (8%).

### 2.3 Analysis of lymphocyte subset and immunomodulatory cells by flow cytometry

To evaluate the effect of deferasirox on immune reconstitution, recovery of lymphocyte subpopulations was assessed by flow cytometry of peripheral blood samples at 1, 3, 6, 9, and 12 months after allo‐HSCT. Mononuclear cells were immunostained with various combinations of the fluorescence‐conjugated antibodies: anti‐CD3 Pacific Blue (clone UCHT1; eBioscience, San Diego, CA), anti‐CD4 APC‐Cy7 (clone OKT4; eBioscience), anti‐CD8 PerCP‐Cy5.5 (clone SK1; eBioscience), and anti‐CD56 FITC (clone TULU56; eBioscience).

Peripheral blood samples from 37 selected patients were further evaluated to reveal the effect of ICT on CD4+CD25+Foxp3+ cells (regulatory T cells). The deferasirox group (n = 29) was chosen according to the following inclusion criteria: at least 2 months duration of deferasirox therapy after allo‐HSCT and sufficient samples at each time points. Eight patients in the nondeferasirox group were chosen based on the presence of elevated serum ferritin level above 1000 ng/mL at 1 month after allo‐HSCT. For intracellular cytokine and Foxp3 staining, surface‐stained cells were processed with fixation and permeabilization buffer (eBioscience) according to the manufacturer's protocol. Prior to intracellular cytokine staining, cells were stimulated in culture medium containing phorbol myristate acetate (25 ng/mL; Sigma‐Aldrich), ionomycin (250 ng/mL; Sigma‐Aldrich, St. Louis, MO, USA), and monensin (GolgiStop, 1 μL/mL; BD Pharmingen, San Jose, CA, USA) in an incubator with 5% CO2 at 37°C for 6 hours. Surface staining was performed with anti‐CD4 FITC (BD555346), anti‐CD25 APC (BD555434), and anti‐Foxp3 PE (BD560046). Flow cytometric analyses were performed on a fluorescence‐activated cell sorting (FACS) Calibur cytometer (BD Pharmingen) using FlowJo software (TreeStar, Ashland, OR).

### 2.4 Statistical analysis

Categorical and continuous variables were compared using the chi‐square test or Fisher's exact test and Student's *t* test or Wilcoxon's rank‐sum test, respectively. OS and disease‐free survival (DFS) curves were plotted using the Kaplan‐Meier method and compared by the log‐rank test. The cumulative incidence was used to estimate the probability of cumulative incidence of relapse (CIR), NRM, and acute GVHD and chronic GVHD, treating nonrelapse death, relapse, and non‐GVHD death as competing risks of relapse, NRM, and each subtype of GVHD, respectively, and compared using the Gray test. For multivariate analysis, variables with a *P*‐value <0.10, as determined by univariate analysis, were considered for entry into the model selection procedure on the basis of the Cox proportional hazards model or a proportional hazards model for a subdistribution of competing risk.^30^ Statistical significance was determined as a *P*‐value ≤0.05 (two‐tailed). For estimating serum ferritin dynamics over time, a repeated measure of ANOVA was used. All statistics were conducted using SPSS, version 13.0 (SPSS, Inc, Chicago, IL), and R‐software (version 3.2.3, R Foundation for Statistical Computing, 2012, http://cran.r-project.org/).

## 3 RESULTS

### 3.1 Clinical impact of hyperferritinemia before and after allo‐HSCT (Cohort 1)

**Figure 2:**
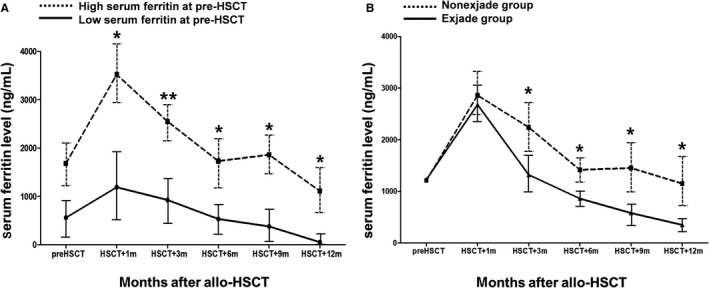
Serial changes in serum ferritin levels before and after allo‐HSCT. Dynamic changes in serum ferritin levels at each time points after allo‐HSCT. (A) cohort 1 (patients not receiving ICT) according to pre‐transplantation hyperferritinemia and (B) cohort 2 (patients who had hyperferritinemia at 1 month after allo‐HSCT) according to deferasirox treatment. Hyperferritinemia was defined as serum ferritin ≥1000 ng/mL. allo‐HSCT, allogeneic hematopoietic stem cell transplantation. *P < 0.05, **P < 0.01

In the first cohort consisting of patients who did not receive deferasirox after allo‐HSCT (n = 198), the median SF level at pre‐transplantation was 1383 ng/mL (range, 132‐12 386 ng/mL) and the high SF group (SF ≥ 1000 ng/mL, n = 104) at pre‐transplantation received significantly more transfusions than low SF group (n = 94). In the high SF group, higher SF level at the time of initial diagnosis (*P* = 0.005) and male predominance (*P* = 0.046) was observed, whereas other characteristics were similar in both the groups (Table 1). The dynamic changes in SF levels after allo‐HSCT revealed that SF level reached the peak level at 1 month followed by a slow decrease (Figure 2A). There were significant differences in SF levels at each time point within a year between the two groups, demonstrating higher SF levels throughout the post‐transplantation period in high SF group at pre‐transplantation.

**Figure 3:**
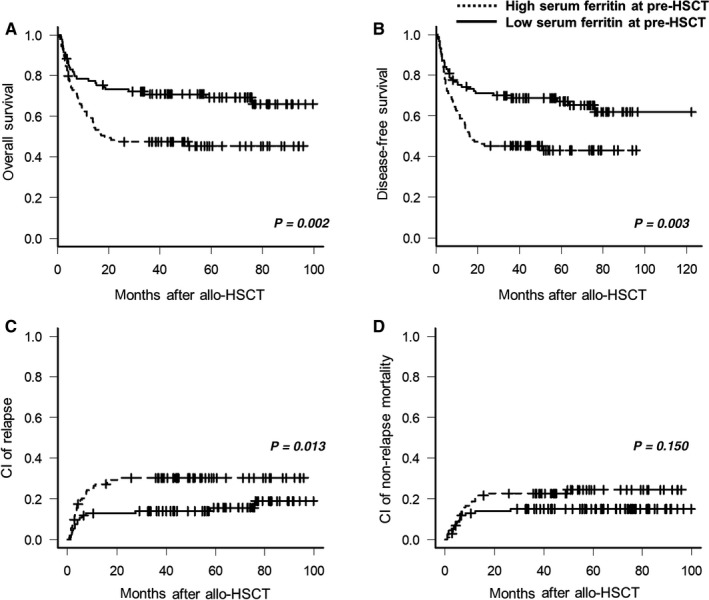
Survival outcomes of cohort 1 according to pre‐transplantation serum ferritin level. The probability of (A) overall survival and (B) disease‐free survival, and (C) cumulative incidence of relapse and (D) nonrelapse mortality in cohort 1 (patients not receiving iron‐chelating therapy). High serum ferritin was defined as ≥1000 ng/mL. allo‐HSCT, allogeneic hematopoietic stem cell transplantation; CI, cumulative incidence

At a median follow‐up of 38 months (range, 1.0‐99.6 months) for survivors, the 4‐year OS, DFS, CIR, and NRM were 60.4% ± 3.2%, 69.5% ± 4.2%, 25.5% ± 7.1%, and 19.7% ± 5.7%, respectively. The high SF group at pre‐transplantation had significantly inferior OS (48.6% vs 71.5%, *P* = 0.002) and DFS (46.6% vs 73.2%, *P* = 0.003) with increased CIR (34.6% vs 16.1%, *P* = 0.013), but no significant difference in NRM (24.4% vs 15.6%, *P* = 0.150) was observed (Figure 3). Multivariate analysis including the factors that were statistically significant in univariate analysis (Table S1) and an adjustment of age, gender, and ELN classification revealed that hyperferritinemia at pre‐transplantation was significantly associated with inferior OS and DFS with increased CIR (Model #1 in Table 2). On the other hand, the two groups did not show any significant difference in the occurrence of acute (29.5% vs 24.4%, *P* = 0.408) and chronic GVHD (38.2% vs 41.4%, *P* = 0.539; Figure S2).

**Table 2:** Multivariate analysis of cohort 1

Factors	OS		DFS		CIR		NRM	
	RR (95% CI)	*P*	RR (95% CI)	*P*	RR (95% CI)	*P*	RR (95% CI)	*P*
Model #1								
SF at pre‐HSCT								
<1000 ng/mL	1		1		1		1	
≥1000 ng/mL	1.97 (1.24‐3.11)	**0.004**	1.87 (1.21‐2.88)	**0.005**	2.09 (1.14‐3.84)	**0.018**	1.45 (0.73‐2.87)	0.280
Age at diagnosis	1.02 (1.00‐1.04)	**0.029**	1.02 (1.01‐1.04)	**0.026**	1.02 (0.99‐1.05)	0.075	1.01 (0.98‐1.04)	0.280
Gender								
Male	1		1		1		1	
Female	1.39 (0.49‐1.42)	0.238	1.23 (0.51‐1.31)	0.268	0.59 (0.32‐1.09)	0.100	1.042 (0.54‐2.03)	0.900
ENL classification								
Good	1		1		1		1	
Int‐1	1.64 (0.69‐3.91)	0.265	0.19 (0.53‐2.34)	0.657	0.89 (0.55‐3.87)	0.740	0.19 (0.10‐2.10)	0.659
Int‐2	1.46 (0.58‐3.72)	0.423	0.89 (0.44‐2.24)	0.343	1.64 (0.14‐4.99)	0.820	1.08 (0.98‐1.25)	0.300
Poor	2.76 (1.13‐6.75)	0.076	4.96 (0.91‐5.10)	0.086	2.59 (0.48‐8.21)	0.441	4.96 (1.29‐5.20)	0.066
Model #2								
SF at post‐HSCT 3 months								
<1000 ng/mL	1		1		1		1	
≥1000 ng/mL	4.71 (1.95‐11.35)	**<0.001**	4.81 (2.09‐11.11)	**<0.001**	6.60 (1.49‐29.14)	**0.013**	3.33 (1.24‐8.98)	**0.017**
Age at diagnosis	1.02 (0.99‐1.04)	0.155	1.02 (0.99‐1.04)	0.119	1.01 (0.98‐1.05)	0.538	1.02 (0.99‐1.05)	0.168
Gender								
Male	1		1		1		1	
Female	0.78 (0.42‐1.44)	0.419	0.89 (0.49‐1.62)	0.708	0.55 (0.22‐1.43)	0.222	0.93 (0.64‐2.76)	0.335
ENL classification								
Good	1		1		1		1	
Int‐1	1.69 (0.48‐5.98)	0.411	0.94 (0.32‐2.76)	0.911	1.09 (0.22‐5.35)	0.917	0.51 (0.33‐3.48)	0.474
Int‐2	1.47 (0.39‐5.43)	0.567	1.04 (0.37‐2.93)	0.942	1.12 (0.22‐5.75)	0.891	1.38 (0.23‐2.99)	0.240
Poor	3.51 (1.00‐12.26)	**0.500**	2.09 (0.75‐5.81)	0.157	1.60 (0.31‐8.35)	0.577	5.98 (0.72‐7.25)	0.074

The bold values represent the statistical significance.

CI, confidence interval; CIR, cumulative incidence of relapse; DFS, disease‐free survival; ENL, European Leukemia Net; HSCT, hematopoietic stem cell transplantation; NRM, nonrelapse mortality; OS, overall survival; RR, relative risk; SF, serum ferritin

In order to analyze the impact of hyperferritinemia after allo‐HSCT, we performed landmark analysis at each time point until 12 months after transplantation. Patients alive at each time point were grouped as low and high SF groups based on the same cut‐off (1000 ng/mL) with pre‐transplantation SF groups. This analysis revealed that hyperferritinemia until 6 months after allo‐HSCT was significantly associated with inferior survival mainly due to increased CIR and thereafter loss of significance mainly due to the selection of relapse‐free survived patients (Table S1). Meanwhile, patients with hyperferritinemia at 3 months after allo‐HSCT also had higher NRM, and multivariate analysis demonstrated that hyperferritinemia at 3 months was significantly associated with inferior OS and DFS with both increased CIR and NRM (Model #2 in Table 2).

### 3.2 Deferasirox after transplantation (Cohort 2)

Among patients with hyperferritinemia at 1 month after allo‐HSCT (n = 276), 46% of patients (consisting of older patients and more haploidentical‐related donor transplants than non‐ICT group) received deferasirox, and haploidentical‐related donor transplants were associated with more reduced intensity conditioning and HLA mismatch.^28^ Other characteristics were similar in both groups (Table 1). Median initiation date of deferasirox was 30 days (range, 28‐50 days) after transplantation, and duration of deferasirox was a median of 5.4 months (range, 1.0‐13.1 months). The starting dose of deferasirox was 20 mg/kg (91%) in AML patients with an exception of 11 patients (9%, 10 mg/kg) due to persistent gastrointestinal symptoms, such as nausea and poor appetite. In the deferasirox group, a faster decline in SF was observed than the nondeferasirox group with significant differences throughout the year (Figure 2B). Dose reduction in deferasirox was required in 64% patients, mainly because of elevation in the levels of creatinine and hepatic enzymes, persistent gastrointestinal disorders, infections, headache, and appearance of skin rash. Adverse events over grade 2 are listed in a Table S2. Deferasirox was finally discontinued in cases achieving optimal range (<500 ng/mL) of SF level (63%) and other events (37%), such as any cause of death (25%), adverse events due to deferasirox administration (4%), and refusal to treatment (8%; Table S3).

### 3.3 Clinical outcomes according to deferasirox (Cohort 2)

**Figure 4:**
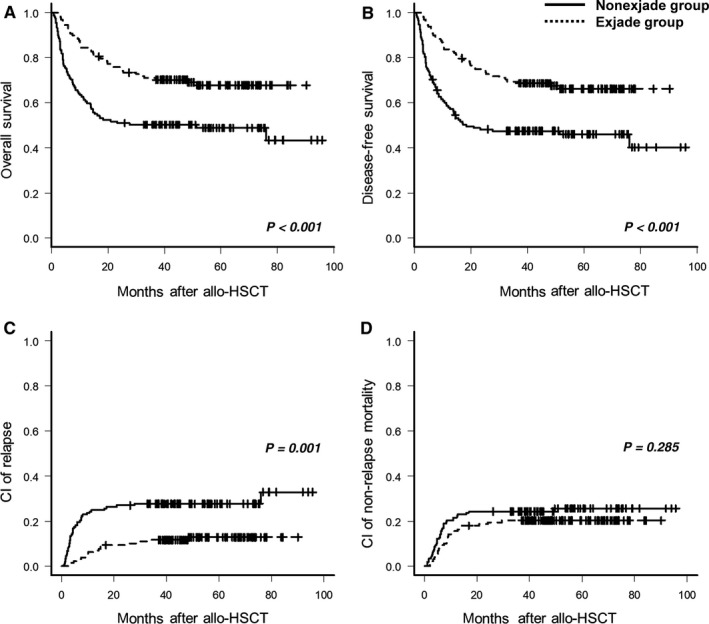
Survival outcomes of cohort 2 according to deferasirox treatment. The probability of (A) overall survival and (B) disease‐free survival, and (C) cumulative incidence of relapse and (D) nonrelapse mortality in cohort 2 (patients who had hyperferritinemia at 1 month after allo‐HSCT). Hyperferritinemia was defined as serum ferritin ≥1000 ng/mL. allo‐HSCT, allogeneic hematopoietic stem cell transplantation; CI, cumulative incidence

At a median follow‐up of 40.7 months (range, 0.8 ‐ 99.6 months) for survivors in the second cohort, the 4‐year OS, DFS, CIR, and NRM were 59.4% ± 2.6%, 57.4% ± 2.7%, 24.5% ± 4.9%, and 21.2% ± 4.9%, respectively. The deferasirox group had significantly superior OS (66.6% vs 50.1%, *P* < 0.001) and DFS (65.4% vs 47.5%, *P* < 0.001) with decrease in CIR (16.5% vs 34.1%, *P* = 0.001) than nondeferasirox group, but no significant difference in NRM was observed (19.1% vs 22.8%, *P* = 0.285; Figure 4). Multivariate analysis including the factors that were statistically significant in univariate analysis (Table S4) and an adjustment of co‐founding factors between deferasirox and nondeferasirox groups revealed a significant association between ICT with deferasirox and superior OS and DFS with decreased CIR (Table 3).

**Table 3:** Multivariate analysis of cohort 2

Factors	OS		DFS		CIR		Chronic GVHD	
	RR (95% CI)	*P*	RR (95% CI)	*P*	RR (95% CI)	*P*	RR (95% CI)	*P*
ICT by deferasirox								
No	1		1		1		1	
Yes	0.45 (0.30‐0.66)	**<0.001**	0.43 (0.29‐0.63)	**<0.001**	0.36 (0.21‐0.64)	**<0.001**	2.52 (1.23‐4.18)	**0.015**
Age at diagnosis	1.02 (1.02‐1.03)	**0.038**	1.02 (1.00‐1.03)	**0.020**	1.03 (1.00‐1.05)	**0.037**	0.99 (0.97‐1.03)	0.750
ENL classification								
Good	1		1		1		—	—
Int‐1	0.51 (0.54‐2.07)	0.473	0.38 (0.21‐0.99)	0.535	0.73 (0.52‐1.92)	0.390		
Int‐2	0.62 (0.52‐2.09)	0.428	0.93 (0.54‐1.98)	0.336	0.91 (0.60‐0.12)	0.932		
Poor	6.57 (0.86‐3.51)	0.051	4.66 (2.91‐ 5.12)	0.131	1.85 (1.28‐3.21)	0.173		
Pre‐HSCT SF								
<1000 mg/dL	1		—	—	—		—	—
≥1000 mg/dL	1.46 (0.97‐2.20)	0.065						
Stem cell source								
BM	—	—	—	—	—	—	1	
PB							2.31 (1.16‐4.59)	**0.017**
Donor type								
Sibling	1		1		1		1	
Unrelated	1.25 (0.69‐1.59)	0.264	1.64 (1.01‐2.01)	0.200	0.27 (0.11‐3.92)	0.605	3.46 (1.99‐5.21)	0.728
Haploidentical	0.12 (0.09‐0.65)	0.735	1.48 (0.00‐1.93)	0.972	0.85 (0.01‐1.15)	0.828	2.81 (0.00‐04.59)	0.382

The bold values represent the statistical significance.

BM, bone marrow; CI, confidence interval; CIR, cumulative incidence of relapse; DFS, disease‐free survival; ELN, European Leukemia Net; GVHD, graft‐vs‐host disease; HSCT, hematopoietic stem cell transplantation; ICT, iron‐chelating therapy; OS, overall survival; PB, peripheral blood; RR, relative risk; SF, serum ferritin.

**Figure 5:**
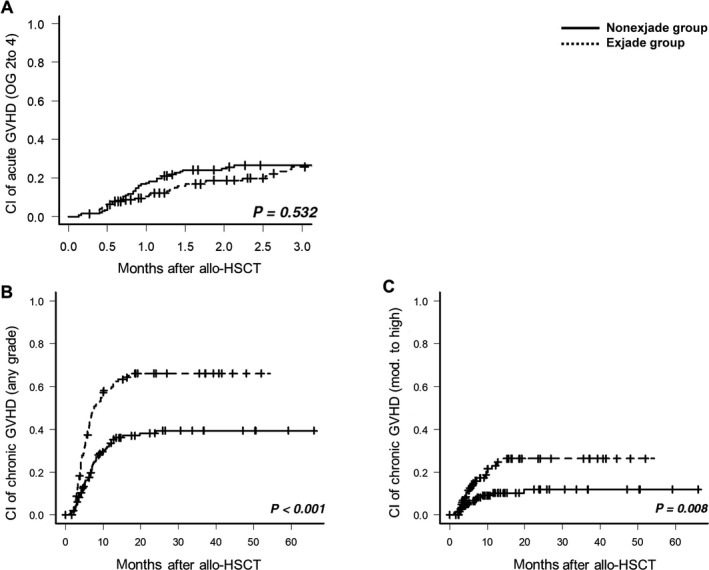
Graft‐vs‐host disease of cohort 2 according to deferasirox treatment. Cumulative incidence of (A) acute GVHD, (B) chronic GVHD with any grade, and (C) chronic GVHD with the moderate and severe grade. allo‐HSCT, allogeneic hematopoietic stem cell transplantation; CI, cumulative incidence

The cumulative incidence of grade II to grade IV of acute GVHD was 26.4% ± 5.5%, and no significant difference (*P* = 0.532) between the deferasirox (27.3% ± 7.4%) and nondeferasirox groups (25.7% ± 8.4%) was observed (Figure 5). Among all the patients with acute GVHD, 11 patients developed grade III to grade IV acute GVHD and the proportion did not differ significantly between the two groups. The 4‐year cumulative incidences of chronic GVHD with mild to severe and moderate to severe were 52.0% ± 6.3% and 18.2% ± 5.5%, respectively (Figure 5). The deferasirox group had significantly higher cumulative incidence of chronic GVHD (mild to severe, 66.1% ± 8.6%; moderate to severe, 26.3% ± 9.5%) than nondeferasirox group (mild to severe, 39.4% ± 8.5%; moderate to severe, 11.9% ± 6.2%; Figure 4). Multivariate analyses revealed that deferasirox was independently associated with the occurrence of chronic GVHD (Table 3).

### 3.4 Effect of deferasirox on immune reconstitution

**Figure 6:**
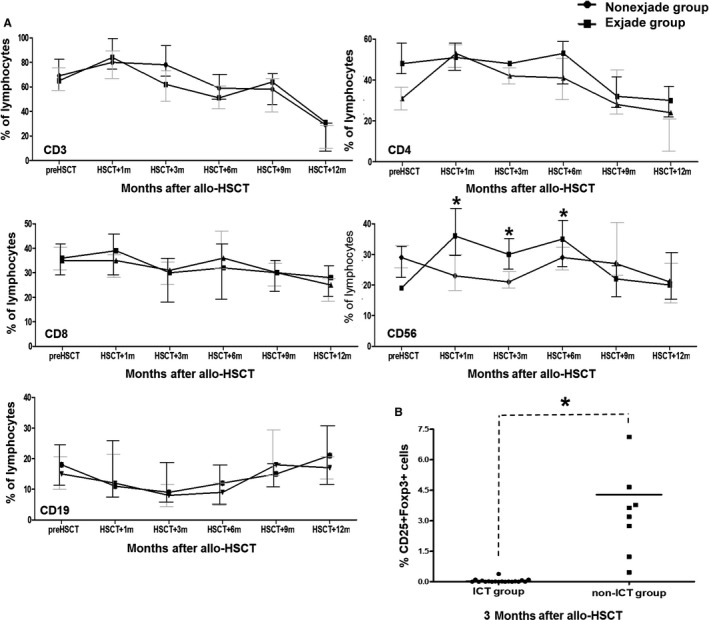
Lymphocyte subsets after allo‐HSCT according to deferasirox treatment. (A) Dynamic changes in lymphocyte subsets at each time points after allo‐HSCT in cohort 2. (B) Regulatory T cells (CD4+CD25+Foxp3+) at 3 months after allo‐HSCT in selected patients (n = 37). allo‐HSCT, allogeneic hematopoietic stem cell transplantation. *P < 0.05, **P < 0.01

Among the patients who survived for at least 6 months without relapse between 1 month and 12 months after allo‐HSCT, post‐transplantation immune reconstitution was analyzed in 82 of 100 patients in the deferasirox group and in 61 of 79 patients in nondeferasirox group, which showed continuation of higher proportion of NK cells in the deferasirox group until 6 months after allo‐HSCT, whereas no significant difference in immune recovery of other subsets was observed (Figure 6A). In an analysis of a separate cohort of 37 patients for regulatory T cells, the ICT group (n = 29) exhibited significantly decreased the proportion of regulatory T cells at 3 months after allo‐HSCT compared to the nondeferasirox group (n = 8; Figure 6B).

## 4 DISCUSSION

Hyperferritinemia due to multiple possible causes, such as iron overload, inflammation, or residual malignancies,^6^ has been associated with poor outcomes after allo‐HSCT.^5^, ^7^, ^8^, ^9^, ^10^, ^11^, ^12^, ^13^, ^14^ Iron overload is a major problem in allo‐HSCT recipients who receive large RBC transfusions during peri‐transplantation periods as well as during the course of chemotherapy in leukemia.^1^, ^2^, ^3^, ^31^ This study revealed the relationship between transfusions during the course of chemotherapy in AML patients and hyperferritinemia before allo‐HSCT, suggesting its reflection of iron overload despite the limit of imperfect nature to define iron overload due to multiple function of SF. The potentials as a biomarker for hyperferritinemia, reflecting inflammation, immune status, and residual leukemia, before and after allo‐HSCT, were clearly demonstrated by the association with inferior survival outcomes in a single disease entity of AML. A therapeutic strategy to reduce SF with deferasirox, an oral iron‐chelating agent having possible antileukemia^20^, ^21^, ^22^ and immune modulatory effects,^23^, ^24^, ^25^ early after allo‐HSCT was tolerable, effectively lowered SF levels and improved survival outcomes compared to a group with hyperferritinemia not receiving deferasirox. The enhanced graft‐vs‐leukemia (GVL) effects in patients receiving deferasirox were found based on the increased cumulative incidence of chronic GVHD and reduced CIR in the deferasirox group, which was supported by the significant suppression of regulatory T cells and sustained higher proportion of NK cells in peripheral blood of deferasirox‐treated patients.

As a promising biomarker to predict outcomes in the setting of allo‐HSCT, the current study confirmed the negative prognostic impact of hyperferritinemia in a single disease entity, AML, in line with previous reports on various hematologic malignancies,^7^, ^8^, ^9^, ^10^, ^11^, ^12^, ^13^, ^14^, ^32^, ^33^ which is in contrast to the failure to reveal association of liver iron contents, the best parameter for iron overload, with allo‐HSCT outcomes in previous studies.^4^, ^5^ Additional potential of hyperferritinemia reflecting inflammation, immune status, and leukemic burden^6^ might contribute this discrepancy as biomarkers in the setting of allo‐HSCT. Recent meta‐analyses suggest that increased NRM might be a main cause of the reduced OS in patients with hyperferritinemia at pre‐transplantation,^15^, ^16^ while some studies showed that it was not associated with increase in NRM.^8^, ^10^, ^13^, ^32^, ^33^ Heterogeneous population of patients in each study could be hypothesized as a major cause for this discrepancy. The current study, including a homogeneous population of AML in remission, distinctly demonstrates that the inferior survival of patients with hyperferritinemia at pre‐transplantation was mainly associated with increases in CIR rather than NRM. In the setting of allo‐HSCT, conditioning‐induced mucositis and release of iron from damaged tissues could raise iron level to undesired levels,^34^ and this possibility is supported by our data showing highest peak of SF level at 1 month after allo‐HSCT followed by a continuous decrease, similar to other reports.^18^, ^19^ However, only a few reports explored in detail the prognostic role of hyperferritinemia after allo‐HSCT on survival,^18^, ^19^ and Meyer et al showed that the hyperferritinemia after allo‐HSCT was associated with an increase in both CIR and NRM.^19^ Our landmark analysis also found the association of hyperferritinemia after allo‐HSCT with inferior survival due to detrimental effects on both CIR and NRM. Despite the limitation of selection bias by the exclusion of dead or relapsed patients before each time point, hyperferritinemia at 3 months after allo‐HSCT demonstrated significant influence in predicting both CIR and NRM, whereas hyperferritinemia at pre‐transplantation only predicted increased risk of CIR. Taken together, this negative prognostic impact of hyperferritinemia at pre‐ and post‐transplantation suggests possible benefit of therapeutic strategies to reduce SF levels after allo‐HSCT.

The potential mechanisms mediating increased mortality in patients with hyperferritinemia at pre‐ and post‐transplantation remain to be unknown. Some studies reported the possibility of iron overload after allo‐HSCT in association with causes of NRM, such as infections, hepatic sinusoidal obstruction syndrome, mucositis, liver dysfunction, and acute GVHD.^2^, ^3^ The toxic effects of hyperferritinemia in the peritransplantation setting may be caused by increased cellular damage due to oxidative stress mediated by non‐transferrin‐bound iron.^34^, ^35^ Meanwhile, our data from patients not receiving deferasirox suggest the association of hyperferritinemia with increased CIR than NRM, which might be related to multiple functions of ferritin, such as cell proliferation, angiogenesis, immunosuppression, and iron delivery.^6^ Indeed, ferritin is known to be differentially overexpressed in tissues in cases of multiple malignancies, as well as SF in patients with various malignancies, often associated with more progressive disease and shorter survival,^6^ while few reports were in hematologic malignancies. In the present study, we observed elevated SF levels in patients with AML even without previous transfusions and concurrent infections at the time of initial diagnosis, indicating the possible relationship between hyperferritinemia and AML. In addition, the immunosuppressive function of ferritin may be related to the increased CIR. Several lines of evidence support the reduced immune response with high SF levels by demonstrating that extracellular ferritin may exert immunosuppressive effects on lymphocytes through modulation of iron delivery.^23^, ^24^, ^36^ Recently, Chen et al demonstrated that iron‐overloaded mice had reduced percentage of T cells and the ratio of helper T‐cell 1/helper T‐cell 2, but increased percentage of regulatory T cells.^25^ Thus, therapeutic strategies for lowering SF levels in the setting of allo‐HSCT could ameliorate aforementioned functions of ferritin and/or iron overload that favor leukemic cell growth and help to elucidate the potential mechanisms mediating increased mortality in patients with hyperferritinemia. However, none of the reported studies has evaluated the impact of iron chelation and/or reduction in SF after allo‐HSCT on outcomes.

In the current study, the deferasirox group was compared with the nondeferasirox group to investigate the therapeutic effect of removing excess iron in patients with hyperferritinemia after allo‐HSCT. In accordance with small prospective trials,^26^, ^27^ our data indicate that management of hyperferritinemia with deferasirox can be safely accomplished after allo‐HSCT in AML patients, even though dose reduction and/or temporary interruption of deferasirox were required in some patients. Creatinine elevation was the most frequent laboratory abnormality observed and the main reason behind dose modification. Despite early initiation of deferasirox (at a median of 30 days after transplantation) with a higher initial dose (20 mg/kg/d), the occurrence of adverse events was similar to the ones presented in the previous reports, in which deferasirox was initiated at 3 or 6 months after allo‐HSCT with a lower initial dose (10 mg/kg/d).^26^, ^27^ In addition, the majority of patients (63%) discontinued the deferasirox with a success of target SF level (<500 ng/mL), suggesting the feasibility of an earlier application of deferasirox in the setting of allo‐HSCT. Of note, the comparison between deferasirox and nondeferasirox group revealed that deferasirox significantly facilitated decline in the SF level and improved survival outcomes, even though the deferasirox group had more high‐risk features, such as more transplants from haploidentical donors. Despite the imperfect balance between the two groups, these results strongly indicate not only the safety and efficacy of ICT by deferasirox, but also reconfirm the detrimental effect of hyperferritinemia after allo‐HSCT.

In this study, the reduced CIR in thedeferasirox group mainly contributed toward the superior survival outcomes to the nondeferasirox group. In terms of functions of SF, ferritin with its high iron storage capacity could serve as a very efficient iron delivery molecule in cancer cells which have high proliferative potential and exhibit higher demand for iron for energy production and DNA synthesis.^6^ Thus, the relative deprivation of iron store by deferasirox could provide the unfavorable environment for the proliferation of leukemic cells, which might contribute to the decrease in CIR. In addition, there are several evidences supporting anti‐leukemic effects of deferasirox by inducing apoptosis and differentiation of AML cells^20^, ^21^, ^22^ and a recent report showed that deferasirox exerts its anti‐leukemia activity by inhibiting ERK phosphorylation.^22^ Another reason for the reduced CIR suggested in this study is the enhanced GVL effects in deferasirox‐treated patients, which were clinically demonstrated by the increased incidence of chronic GVHD. Chronic GVHD is well known to be related to GVL effects.^37^, ^38^ We revealed sustained higher proportion of NK cells and profoundly suppressed regulatory T cells in deferasirox‐treated patients, which suggests possible contribution to the enhanced GVL effects despite the limitation of selecting the samples for analysis of immune subsets in a small subset of patients. Our data and aforementioned evidence for the immunosuppressive function of ferritin and/or iron overload^23^, ^24^, ^25^ suggest that deferasirox might reverse immunosuppression by hyperferritinemia, which could enhance the GVL effect in the setting of allo‐HSCT. The detailed underlying mechanisms of deferasirox‐induced decrease in CIR in the setting of allo‐HSCT should be further explored.

In conclusion, the present study demonstrates the negative prognostic role of hyperferritinemia not only before but also after allo‐HSCT mainly through the compromised GVL effects. Deferasirox treatment was feasible and beneficial even when initiated early after allo‐HSCT. An increase in the occurrence of chronic GVHD with suppressed regulatory T cells and sustained higher proportion of NK cells in the deferasirox‐treated patients indicates restoration of GVL effects, resulting in the reduced CIR and better survival despite more high‐risk features of deferasirox group. To the best of our knowledge, this is the first study to show the impact of deferasirox administration in lowering SF levels and on survival after allo‐HSCT, despite the presence of some limitations of retrospective nature and imperfect balance between the two groups. In future, controlled and randomized studies would be of importance to confirm the benefits of deferasirox after allo‐HSCT.

## CONFLICT OF INTEREST

The authors declare that they have no personal or financial conflict of interest.
