# Supplementary material for: Improved survival outcomes and restoration of graft‐vs‐leukemia effect by deferasirox after allogeneic stem cell transplantation in acute myeloid leukemia
Source: Cancer Med. 2019 Jan 24;8(2):501–14. doi: 10.1002/cam4.1928 (PMC6382990; doi:10.1002/cam4.1928)
Supplement: Supplementary file 1 [file CAM4-8-501-s001.doc]

**Supplementary**-Table 1. Univariate analysis of cohort 1

| **Factors** | **OS** | | **DFS** | | **CIR** | | **NRM** | |
| --- | --- | --- | --- | --- | --- | --- | --- | --- |
|  | RR (95%CI) | *P* | RR (95%CI) | *p* | RR (95%CI) | *P* | RR (95%CI) | *p* |
| **Age at diagnosis** | 1.59 (1.01-2.04) | **0.042** | 1.02 (1.00-1.04) | **0.013** | 1.39 (0.99-1.44) | 0.270 | 1.02 (0.99-1.04) | 0.115 |
| **Gender (male)** | 0.70 (0.46-1.08) | 0.107 | 0.74 (0.49-1.13) | 0.163 | 1.11 (0.31-1.30) | 0.740 | 1.03 (0.57-1.85) | 0.932 |
| **ENL classification**  good  int-1  int-2  poor | 1  1.72 (0.72-4.09)  1.70 (0.68-4.29)  2.64 (1.09-6.42) | 0.221  0.259  **0.032** | 1  1.23 (0.59-2.56)  1.19 (0.54-2.63)  1.75 (0.82-3.74) | 0.578  0.669  0.148 | 1  1.20 (0.45-3.22)  1.28 (0.45-3.70)  1.37 (0.47-3.94) | 0.590  0.789  0.110 | 1  1.35 (0.45-4.00)  1.18 (0.36-3.91)  2.46 (0.827.42) | 0.590  0.789  0.110 |
| **Pre-HSCT SF**  < 1,000 ng/ml  ≥ 1,000 ng/ml | 1  2.04 (1.30-3.21) | **0.002** | 1  1.95 (1.26-3.00) | **0.003** | 1  2.19 (1.18-4.07) | **0.013** | **1**  **1.75 (0.95-3.22)** | **0.072** |
| **Post-HSCT SF at1 month***  < 1,000 ng/ml  ≥ 1,000 ng/ml | 1  3.68 (1.93-7.78) | **<0.001** | 1  3.38 (1.87-6.71) | **<0.001** | 1  4.21 (1.51-11.76) | **0.002** | 1  2.35 (0.93-5.98) | 0.091 |
| **Post-HSCT SF at 3 months***  < 1,000 ng/ml  ≥ 1,000 ng/ml | 1  3.90 (1.83-8.30) | **<0.001** | 1  4.63 (1.98-8.38) | **<0.001** | 1  6.06 (1.41-26.01) | **0.005** | 1  2.95 (1.02-8.55) | **0.029** |
| **Post-HSCT SF at 6 months***  < 1,000 ng/ml  ≥ 1,000 ng/ml | 1  3.69 (1.34-10.19) | **0.007** | 1  3.32 (1.29-8.51) | **0.008** | 1  8.07 (1.36-48.09) | **0.020** | 1  2.06 (0.68-6.26) | 0.215 |
| **Post-HSCT SF at 9 months***  < 1,000 ng/ml  ≥ 1,000 ng/ml | 1  3.24 (0.93-10.63) | **0.048** | 1  2.63 (0.85-8.17) | 0.083 | 1  6.04 (0.90-40.47) | 0.071 | 1  1.82 (0.39-8.53) | 0.422 |
| **Post-HSCT SF at 12 months***  < 1,000 ng/ml  ≥ 1,000 ng/ml | 1  2.56 (0.81-8.04) | 0.096 | 1  2.20 (0.73-6.66) | 0.151 | 1  2.24 (0.35-14.43) | 0.401 | 1  2.26 (0.59-8.52) | 0.239 |
| **Stem cell source**  BM  PB | 1  1.22 (0.79-1.87) | 0.373 | 1  1.28 (0.84-1.94) | 0.239 | 1  1.64 (0.92-2.92) | 0.096 | 1  0.97 (0.53-1.77) | 0.927 |
| **ATG usage**  No  Yes | 1  1.16 (0.74-1.83) | 0.520 | 1  1.17 (0.75-1.81) | 0.490 | 1  1.35 (0.74-2.46) | 0.327 | 1  0.98 (0.51-1.88) | 0.953 |
| **Donor type**  sibling  unrelated  haploidentical | 1  1.31 (0.85-2.04)  0.95 (0.30-3.05) | 0.226  0.929 | 1  1.34 (0.88-2.05)  0.88 (0.28-2.83) | 0.173  0.833 | 1  1.19 (0.65-2.18)  1.10 (0.26-4.64) | 0.563  0.897 | 1  1.52 (0.84-2.77)  0.63 (0.09-4.67) | 0.169  0.652 |
| **Conditioning intensity**  myeloablation  reduced intensity | 1  1.07 (0.66-1.71) | 0.795 | 1  1.15 (0.73-1.81) | 0.540 | 1  1.36 (0.74-2.49) | 0.327 | 1  0.86 (0.43-1.70) | 0.665 |
| **Conditioning regimen**  TBI based  Non-TBI based | 1  1.23 (0.69-2.18) | 0.481 | 1  1.24 (0.71-2.15) | 0.452 | 1  1.50 (0.72-3.11) | 0.277 | 1  1.01 (0.43-2.38) | 0.987 |
| **HLA matched status**  match  mismatch | 1  1.52 (0.90-2.56) | 0.118 | 1  1.51 (0.91-2.45) | 0.114 | 1  1.72 (0.87-3.39) | 0.117 | 1  1.27 (0.59-2.73) | 0.544 |
| **Donor ABO match**  match  mismatch | 1  0.81 (0.53-1.25) | 0.348 | 1  0.75 (0.49-1.14) | 0.179 | 1  0.73 (0.40-1.32) | 0.298 | 1  0.77 (0.42-1.40) | 0.385 |
| **Infused stem cell counts**  < 5 x 10^6^/kg  ≥ 5 x 10^6^/kg | 1  1.52 (0.98-2.33) | 0.062 | 1  1.51 (0.99-2.29) | 0.053 | 1  1.56 (0.87-2.78) | 0.130 | 1  1.22 (0.63-2.34) | 0.550 |

* Landmark analysis at each time point after transplantation with patients who were alive without relapse at each time point without relapse. **Abbreviation**: ATG, Anti-thymocyte globulin; BM, bone marrow; CI, confidence interval; CIR, cumulative incidence of relapse; DFS, disease-free survival; ELN, European Leukemia Net; GVHD, Graft-versus-host disease; HLA, human leukocyte antigen; HSCT, hematopoietic stem cell transplantation; ICT, iron-chelating therapy; NRM, non-relapse mortality; OS, overall survival; PB, peripheral blood; RR, relative risk; SF, serum ferritin; TBI, total body irradiation.

**Supplementary-Table 2. Drug-related adverse events in deferasirox group of cohort 2 (n=128)**

| **Adverse events (over grade 2)** | **n (%) *** |
| --- | --- |
| abdominal discomfort | 22 (17.2) |
| nausea | 19 (14.8) |
| vomiting | 15 (11.7) |
| loose stool | 28 (21.9) |
| rash or urticaria | 16 (12.5) |
| headache | 22 (17.2) |
| eye discomfort | 3 (2.3) |
| fatigue | 6 (4.7) |
| increased serum creatinine level | 41 (32.0) |
| elevated liver enzyme | 12 (9.4) |

* The number of patients was selected in duplicate.

**Supplementary-Table 3. The cause of derasirox-discontinuation in cohort 2 (n=128)**

| **Cause** | **No. of patient** (%) | **Duration of deferasirox-use**  , median month (range) | **SF level at cessation**  **of deferasirox, median** (range) |
| --- | --- | --- | --- |
| Success of optimal ferritin level (<500 ng/ml) | 81 (63.3) | 8.9 (4.0-12.1) | 428 (325-500) |
| Adverse event due to ICT alone | 5 (3.9) | 4.0 (3.7-4.2) | 927 (891-910) |
| Any cause of death | 32 (25.0) | 5.1 (1.0-13.1) | 1,820 (598-7895) |
| Patients refusal | 10 (7.8) | 5.3 (2.0-9.9) | 1,045 (790-1650) |

**Abbreviation**: SF, serum ferritin.

| **Factors** | **OS** | | **DFS** | | **RI** | | **Chronic GVHD** | |
| --- | --- | --- | --- | --- | --- | --- | --- | --- |
|  | RR(95%CI) | *p* | RR(95%CI) | *p* | RR(95%CI) | *p* | RR(95%CI) | *p* |
| **Age at diagnosis** | 1.01 (0.99-1.03) | 0.074 | 1.38 (0.99-1.03) | 0.090 | 1.02 (0.99-1.04) | 0.077 | 1.00 (0.98-1.03) | 0.803 |
| **Gender (male)** | 0.88 (0.61-1.27) | 0.493 | 0.94 (0.56-1.68) | 0.714 | 0.72 (0.42-1.24) | 0.236 | 0.88 (0.46-1.68) | 0.694 |
| **ENL classification**  good  int-1  int-2  poor | 1  1.04 (0.54-2.02)  1.13 (0.56-2.26)  1.89 (0.95-3.79) | 0.903  0.730  0.070 | 1  0.92 (0.50-1.69)  0.95 (0.49-1.81)  1.54 (0.81- 2.96) | 0.777  0.867  0.191 | 1  1.01 (0.36-2.21)  0.79 (0.47-3.00)  1.62 (0.61-4.09) | 0.806  0.724  0.357 | 1  1.48 (0.43-5.05)  1.77 (0.49-6.27)  1.26 (0.31-5.03) | 0.532  0.377  0.747 |
| **ICT by deferasirox**  no  yes | 1  0.47 (0.32-0.69) | **<0.001** | 1  0.46 (0.32-0.67) | **<0.001** | 1  0.35 (0.19-0.62) | **<0.001** | 1  1.88 (0.97-3.64) | **0.002** |
| **SF at pre-HSCT**  < 1,000 ng/ml  ≥ 1,000 ng/ml | 1  1.53 (1.02-2.30) | **0.039** | 1  1.28 (0.95-2.07) | 0.089 | 1  1.59 (0.89-2.83) | 0.115 | 1  0.51 (0.26-0.96) | **0.036** |
| **Stem cell source**  BM  PB | 1  1.00 (0.69-1.45) | 0.991 | 1  0.98 (0.69-1.40) | 0.907 | 1  1.30 (0.77-2.20) | 0.327 | 1  2.31 (1.16-4.60) | **0.017** |
| **ATG usage**  No  Yes | 1  1.03 (0.71-1.51) | 0.86 | 1  1.04 (0.72-1.50) | 0.831 | 1  1.02 (0.60-1.74) | 0.931 | 1  1.17 (0.61-2.24) | 0.642 |
| **Conditioning regimen**  TBI based  Non-TBI based | 1  1.32 (0.78-2.24) | 0.304 | 1  1.33 (0.79-2.23) | 0.272 | 1  1.51 (0.74-3.07) | 0.258 | 1  2.20 (0.96-5.01) | 0.060 |
| **HLA matched status**  match  mismatch | 1  1.15 (0.76-1.75) | 0.507 | 1  1.11 (0.4-1.67) | 0.618 | 1  1.13 (0.63-2.04) | 0.678 | 1  1.10 (0.38-1.60) | 0.625 |
| **RBC transfusion (unit)**  < 32  ≥ 32 | 1  1.30 (0.66-2.59) | 0.456 | 1  1.36 (0.71-2.59) | 0.352 | 1  1.17 (0.42-3.24) | 0.762 | 1  0.94 (0.29-3.08) | 0.924 |
| **SDP transfusion (unit)**  < 14  ≥ 14 | 1  0.89 (0.61-1.30) | 0.552 | 1  0.89 (0.61-1.29) | 0.531 | 1  1.26 (0.71-2.25) | 0.428 | 1  0.56 (0.29-1.06) | 0.075 |
| **Donor ABO match**  well match  mismatch | 1  0.76 (0.52-1.10) | 0.149 | 1  0.74 (0.52-1.07) | 0.107 | 1  0.70 (0.41-1.19) | 0.184 | 1  0.76 (0.39-1.45) | 0.405 |
| **Infused stem cell counts**  < 5 x 10^6^/kg  ≥ 5 x 10^6^/kg | 1  1.06 (1.00-1.11) | 0.064 | 1  1.06 (1.00-1.11) | 0.071 | 1  1.08 (0.92-2.01) | 0.616 | 1  1.05 (0.96-1.15) | 0.260 |

**Supplementary-Table 4. Univariate analysis of cohort 2 (n=276)**

**Abbreviation**: ATG, Anti-thymocyte globulin; BM, bone marrow; DFS, disease-free survival; ELN, European Leukemia Net; GVHD, graft-versus-host disease; HLA, human leukocyte antigen; HSCT, hematopoietic stem cell transplantation; ICT, iron-chelating therapy; OS, overall survival; PB, peripheral blood; RBC, red blood cells; RI, relapse incidence; RR, relative risk; SDP, single donor platelet; SF, serum ferritin; TBI, total body irradiation.

**Supplementary-Figure 1. Serial change of serum C-reactive protein levels before and after alloHSCT**


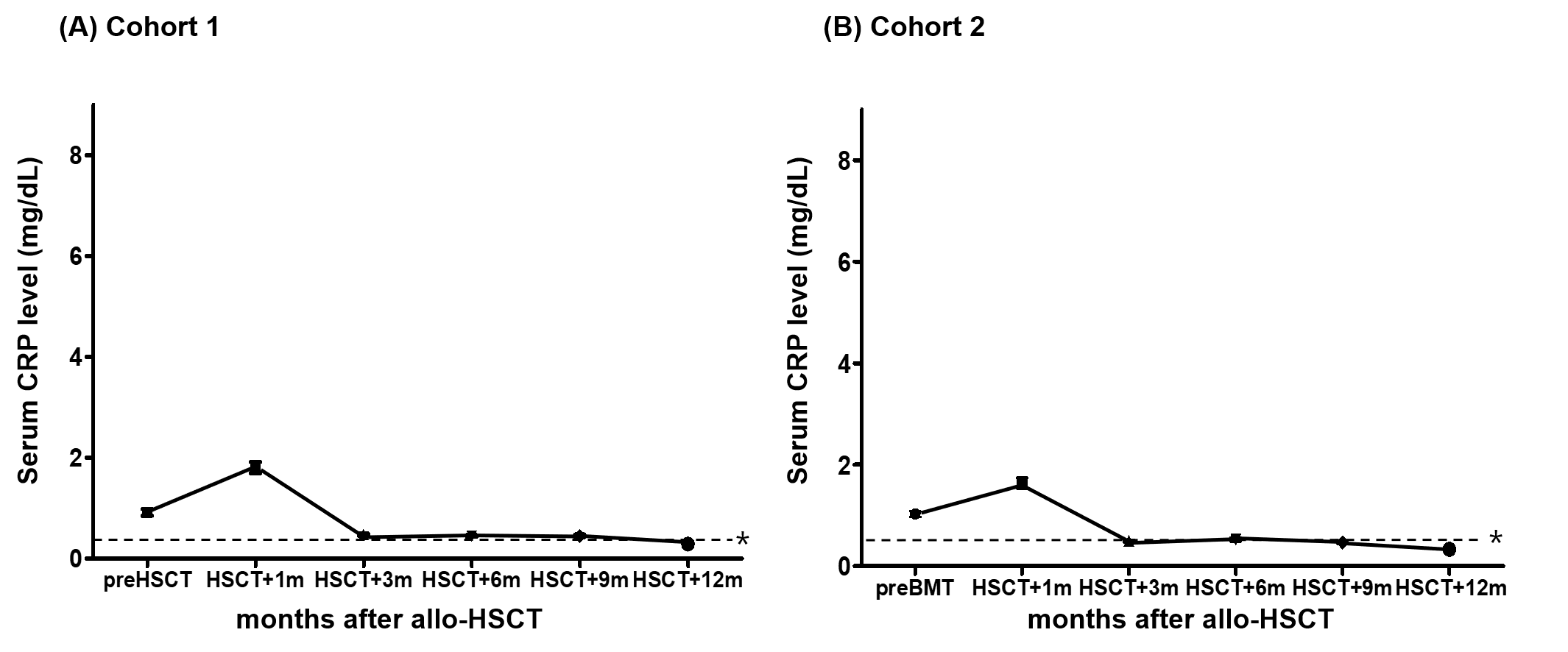


* Reference range of serum CRP is below 0.5 mg/dL

**Supplementary-Figure 2. Graft versus host disease of Cohort 1**

**
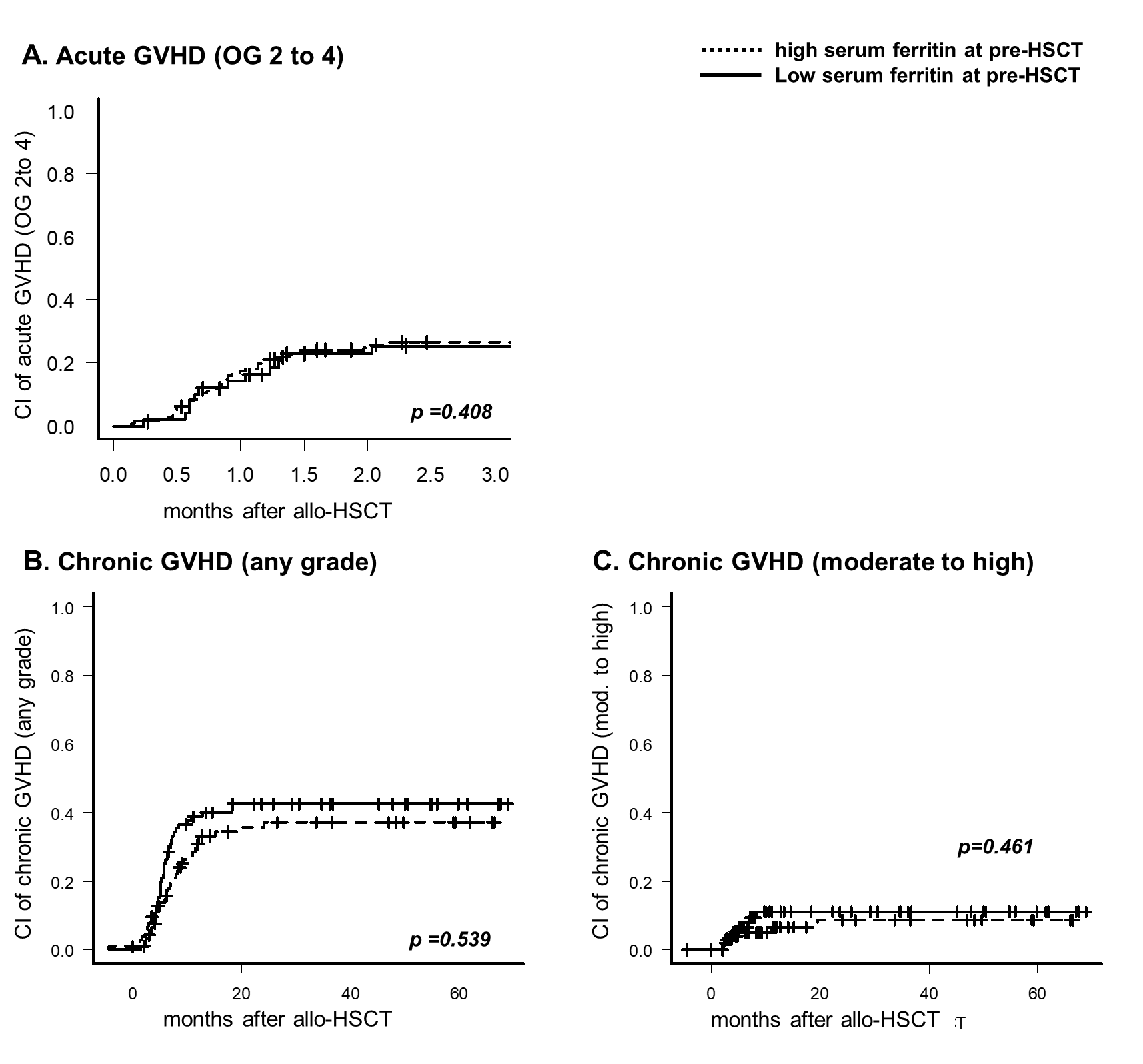
**
